# Whole-genome sequencing of a *Janthinobacterium sp*. isolated from the Patagonian Desert

**DOI:** 10.1128/mra.00600-24

**Published:** 2024-10-09

**Authors:** Nicole T. Cavanaugh, Girish Kumar, Alicyn Reverdy Pearson, Julia Colbert, Carlos Riquelme, André O. Hudson, Yunrong Chai, Veronica Godoy-Carter

**Affiliations:** 1 Department of Biology, Northeastern University College of Science, Boston, Massachusetts, USA; 2 Rochester Institute of Technology College of Science, Thomas H. Gosnell School of Life Sciences, Rochester, New York, USA; 3 Departamento de Biotecnología, Universidad de Antofagasta, Antofagasta, Chile; DOE Joint Genome Institute, Berkeley, California, USA

**Keywords:** environmental microbiology, soil microbiology, genomics, extremophiles

## Abstract

*Janthinobacterium* is a genus of Gram-negative environmental bacteria that survive extreme conditions by forming biofilms and producing pigments. *Janthinobacterium* sp. LS2A, an extremophile isolated from soil in the Chilean Patagonia, contains seven known biosynthetic gene clusters, including the purple pigment violacein, which may aid in its survival in harsh environments.

## ANNOUNCEMENT

*Janthinobacterium* is a genus of rod-shaped Gram-negative bacteria that is widespread in cold environments (1–3). Some produce a purple pigment called violacein (4). Violacein is of high biotechnological significance due to its known antiviral, antibacterial, antimycotic, antitumor, algicidal, and antioxidant activities (5–8). The genus also contains extremophiles that are tolerant to temperatures 2%–28°C and UV exposure (9–11). *Janthinobacterium sp*. also form biofilms, which help them survive these harsh conditions (4).

**Fig 1:**
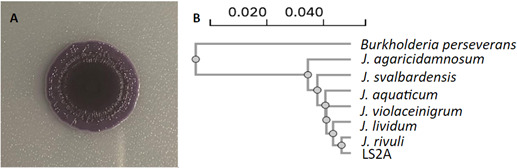
*Janthinobacterium sp*. LS2A forms biofilms, and its sequence demonstrates it belongs to the genus. (A) Biofilm grown from *Janthinobacterium sp.* LS2A. (B) Phylogenetic tree. A phylogenetic tree relating the LS2A V3/4 16S rRNA regions to other *Janthinobacterium sp*. in the RefSeq database. From top to bottom: *Burkholderia preserverans* NR_179094.1, *J. agaricidamonosum* NR_114134.1, *J. svalbardensis* NR_132608.1, *J. aquaticum* NR_170539.1, *J. violaceinigrum* NR_170541.1, *J. lividum* NR_026365.1, *J. rivuli* NR_170540.1, and *Janthinobacterium sp.* LS2A.

The goal of this project was to study extremophiles in Patagonia and the mechanisms that help them survive in extreme environments. Soil from Laguna Sarmiento (51.0636 S; 72.9264 W) was collected in July 2017. Bacteria were isolated after plating 100 µL of a soil/water slurry on Reasoner’s 2A (R2A) agar plates, followed by incubation at 28°C for 48–72 hours. Several bacterial colonies emerged; a single purple colony was picked and assigned the name “LS2A.” To test biofilm formation, LS2A was grown at 30°C to OD600 = 1.0 in R2A broth, and 2 µL of the culture was spotted on a dry R2A plate and incubated for 96 hours before imaging (Fig. 1A). As a first approximation to identify closely related species, we performed 16S rRNA gene sequencing on the V3/4 variable regions using the following primers: 5′-CCTACGGGNGGCWGCAG-3′ and 5′-GACTACHVGGGTATCTAATCC-3′. The isolate was identified as *Janthinobacterium sp*. using the National Society for Biotechnology Information (NCBI) BLASTN version 2.15.0 (12, 13). The sequences of the V3/4 16S rRNA region, along with a variety of 16S sequences from *Janthinobacterium* sp. from the RefSeq database, were used to create a multiple sequence alignment and guide tree using ClustalOmega version 1.2.4 (Fig. 1B) (14).

Genomic DNA was isolated from a 2-mL R2A liquid culture grown at 30°C overnight using the Wizard Genomic DNA Purification Kit (Promega, USA) following the manufacturer’s instructions for Gram-negative bacteria. DNA libraries were prepared using the Nextera XT library preparation kit (Illumina) and sequenced using the Illumina MiSeq System at the Genomics Lab, Rochester Institute of Technology. Raw paired-end reads were trimmed using Trimmomatic Galaxy version 0.38.1 (15). The trimmed reads were assembled *de novo* using Unicycler Galaxy version 0.5.0+galaxy1 (16). Quality analysis was performed using Quast Galaxy version 5.2.0+galaxy1 (17) and FastQC Galaxy version 0.74 + galaxy0 (18). All programs listed above were run using default parameters unless stated otherwise. The assembly consists of 4,575,745 reads totaling 667.6 million base pairs (Mbp). These reads assembled into 29 contigs with a total length of 6,251,303 base pairs (bp). The estimated genome coverage is 105 x with 98.45% completeness. The N50 is 540,701 bp, and the GC content is 62.69%. Analysis by the Prokaryotic Genome Analysis Pipeline version 6.6 revealed that the LS2A genome contained 5,664 total genes, 5,548 protein-coding sequences, three rRNAs operons, and 77 tRNAs (19–21).

A summary of the biosynthetic gene clusters and secondary metabolites predicted by antibiotics and secondary metabolite analytics shell (antiSMASH) version 7.1.0 is outlined in Table 1 (22). antiSMASH detected seven gene clusters that encode unique metabolites, including violacein.

**TABLE 1:** Predicted BGCs in LS2A^*a*^

Region	Type	Start	End	Most Similar To	Similarity Score
1.1	RiPP-like	364,750	372,021	Burkholderic acid	Low (<10%)
1.2	Indole	533,201	556,218	Violacein	100%
1.3	RiPP-like	830,949	841,878		
1.4	Acyl amino acids	936,131	1,031,826	O-antigen	14%
1.5	Terpene	1,087,968	1,109,729		
3.1	RiPP-like	254,757	266,376		
7.1	Arylpolyene	1	32,414	APE Ec	36%

^*a*^ A summary of biosynthetic gene clusters (BGCs) in *Janthinobacterium sp*. LS2A identified by antiSMASH.

## Data Availability

The WGS projects for *Janthinobacterium sp.* LS2A have been deposited in GenBank under BioProject number PRJNA1071647, BioSample number SAMN39706722, and SRA number SRR27839980. Results from PGAP analysis can be found using accession number JAZHPB000000. The following sequences were used to construct Fig. 1 and were retrieved from GenBank: NR_179094.1, NR_114134.1, NR_132608.1, NR_170539.1, NR_170541.1, NR_026365.1, and NR_170540.1. The NCBI BLASTN webserver was used to identify the LS2A 16s rRNA gene sequence. All programs found on the Galaxy platform were run using Galaxy version 23.1 and can be found at usegalaxy.org. Clustal Omega can be found on the EMBL European Bioinformatics Institute webpage.
